# Impact of a structured nurse-delivered multi component SGLT2 inhibitor initiation and optimization pathway on kidney function and clinical outcomes in type 2 diabetes and chronic kidney disease: a real-world retrospective study

**DOI:** 10.1186/s12882-026-04842-z

**Published:** 2026-03-14

**Authors:** Liang Huang, Muhui Yi

**Affiliations:** 1 Clinical Pharmacy Center, Nanfang Hospital, Southern Medical University, Guangdong, Guangzhou China; 2 Huiqiao Medical Center, Nanfang Hospital, Southern Medical University, Guangdong, Guangzhou China

**Keywords:** SGLT2 inhibitors, Care bundle, Type 2 diabetes mellitus, Chronic kidney disease, Real-world evidence, Implementation strategy

## Abstract

**Background:**

The implementation gap between evidence-based SGLT2 inhibitor therapy and real-world practice remains substantial in patients with type 2 diabetes mellitus (T2DM) and chronic kidney disease (CKD). Protocolized care delivery bundles incorporating frequent follow-up, systematic monitoring and adherence support may bridge this implementation gap.

**Objective:**

To evaluate the impact of a a structured, nurse-delivered multi component pathway (frequent contact, protocolized monitoring, and adherence reminders) on kidney function progression and clinical outcomes in patients with T2DM and CKD.

**Methods:**

This single-center, retrospective cohort study included 450 patients with T2DM and CKD who initiated SGLT2 inhibitors between February 2024 and February 2025. Patients were categorized into structured nurse-delivered multi component pathway management (*n* = 225) or conventional care (*n* = 225) groups. Primary outcomes included annualized eGFR decline rate and composite kidney endpoint (≥ 40% eGFR decline, end-stage kidney disease, or kidney-related death). Propensity score weighting was applied to balance baseline characteristics.

**Results:**

The structured nurse-delivered multi component group demonstrated a slower annualized eGFR decline compared to conventional care. The composite kidney endpoint occurred less frequently in the structured nurse-delivered multi component group (HR 0.68, 95% CI 0.52–0.89, *P* = 0.004). Laboratory follow-up compliance was significantly higher in the structured nurse group (82.3% vs. 64.7%, *P* < 0.001). Adverse event-related drug discontinuation was lower with structured nurse-delivered multi component group (8.4% vs. 15.2%, *P* = 0.012).

**Conclusion:**

A structured SGLT2 inhibitor management bundle delivered through a nurse-coordinated pathway reflecting greater care intensity and standardized follow-up was associated with improved kidney function trajectories and fewer adverse kidney outcomes in routine practice.

**Clinical trial number:**

The clinical trial was registered in the Chinese Clinical Trial Registry (ChiNHSMU20231015).

**Supplementary Information:**

The online version contains supplementary material available at 10.1186/s12882-026-04842-z.

## Introduction

Diabetic kidney disease (DKD) represents a leading cause of end-stage kidney disease globally and significantly increases cardiovascular mortality risk. Despite conventional therapies including renin-angiotensin-aldosterone system (RAAS) inhibitors, substantial residual risk persists in patients with type 2 diabetes mellitus (T2DM) and chronic kidney disease (CKD) [1, 2]. The approval of sodium-glucose cotransporter-2 (SGLT2) inhibitors for CKD treatment marked a pivotal shift in CKD management, with subsequent approvals of dapagliflozin and empagliflozin for patients with CKD both with and without T2DM [3].

Major clinical trials including DAPA-CKD and EMPA-KIDNEY have demonstrated that SGLT2 inhibitors significantly reduce the risk of kidney disease progression, with DAPA-CKD showing a 39% relative risk reduction in the composite outcome of sustained eGFR decline, end-stage kidney disease, or cardiovascular death [4, 5]. The EMPA-KIDNEY trial confirmed similar efficacy with empagliflozin, demonstrating a 28% relative risk reduction in kidney disease progression across a broad CKD population [3, 6]. These landmark trials have established SGLT2 inhibitors as foundational therapy for cardiorenal protection in DKD [7].

However, the uptake of SGLT2 inhibitors has been slow, especially among patients without T2DM, owing to lack of certainty and familiarity among healthcare professionals [8, 9]. The “last mile” challenge in translating trial evidence into clinical practice involves multiple barriers including inconsistent initiation protocols, inadequate patient education regarding volume management and sick-day rules, suboptimal laboratory monitoring schedules, and variable dose adjustment practices [10]. Testing for CKD with urinary albumin-to-creatinine ratio and eGFR in adults with diabetes and hypertension remains low in routine clinical care, further compounding the implementation gap [11–13].

Structured nurse-delivered multi component group interventions have demonstrated beneficial outcomes in ameliorating diabetes management through structured algorithms for care and enhanced patient education [14, 15]. Meta-analyses have shown that structured nurse-delivered multi component group diabetes self-management education programs improve long-term glycemic control and self-efficacy, offering scalable and cost-effective models for chronic disease management [16, 17]. The advantages of nursing teams in chronic disease management include: (1) process standardization through protocol-driven care, (2) enhanced medication adherence through intensive patient education, (3) early identification and graded management of adverse events, and (4) facilitation of multidisciplinary care coordination.

Importantly, nurse delivered pathways often represent bundled implementation strategies rather than a single-agent intervention. In this study, the pathway integrates more frequent patient contact, protocolized laboratory monitoring, and adherence support/reminder systems alongside standardized education. Accordingly, our primary comparison evaluates the association of this multi component care delivery model with kidney outcomes among patients initiating SGLT2 inhibitors, rather than isolating the independent causal effect of ‘nurse-developed’ care.

The objectives of this study were to:

1. Evaluate the impact of a structured nurse-delivered multi component group SGLT2 inhibitor initiation and dose optimization pathway on annualized eGFR decline rate in patients with T2DM and CKD.
2. Assess the effect on composite kidney endpoints including sustained eGFR decline, progression to end-stage kidney disease, and kidney-related mortality.
3. Compare adverse event rates and medication adherence between structured nurse-delivered multi component group and conventional management approaches.
4. Identify implementation factors associated with successful SGLT2 inhibitor therapy optimization. achievement.

## Methods

### Study design

This single-center, retrospective cohort study utilized electronic medical record (EMR) and nursing information system (NIS) data from a tertiary hospital with integrated endocrinology-nephrology clinics. The study employed a real-world design comparing patients managed through a structured nurse-delivered multi component group SGLT2 inhibitor pathway versus conventional care. The baseline date was defined as the date of first SGLT2 inhibitor prescription or enrollment into the structured nurse-delivered multi component group pathway between February 1, 2024, and February 28, 2025. The observation period extended from baseline through at least 6 months of follow-up or until outcome occurrence or censoring. This retrospective analysis was conducted according to principles outlined in the Strengthening the Reporting of Observational Studies in Epidemiology (STROBE) guidelines for cohort studies.

The study was approved by the Ethics Committee of Nanfang Hospital, Southern Medical University, Baiyun District, Guangzhou, China (Ethics Review No. 202312023). All procedures involving human participants were performed in accordance with the ethical standards of the institutional and/or national research committee and with the 1964 Declaration of Helsinki and its later amendments. Written informed consent was obtained from all individual participants included in the study.

### Study setting and management model overview

The integrated endocrinology-nephrology clinic at our institution serves as a unified platform for managing patients with diabetes and kidney disease. Within this framework, a structured structured nurse-delivered multi component group SGLT2 inhibitor management pathway was implemented, encompassing: (1) standardized baseline assessment protocols, (2) individualized dose initiation and titration algorithms, (3) stratified laboratory monitoring schedules, (4) telephone and digital follow-up systems, and (5) tiered adverse event reporting and management mechanisms. The pathway was developed collaboratively by endocrinologists, nephrologists, clinical pharmacists, and diabetes specialist nurses, with clearly defined roles and decision-making authorities. Patients in the intervention group received comprehensive SGLT2 inhibitor management through this structured pathway, while the comparison group received standard care delivered through conventional physician-led outpatient visits without systematic nursing protocol implementation.

### Study population

#### Inclusion criteria

Eligible participants met all of the following criteria: (1) age ≥ 18 years with confirmed diagnosis of T2DM according to American Diabetes Association criteria, (2) presence of CKD defined by estimated glomerular filtration rate (eGFR) < 90 mL/min/1.73 m² and/or urinary albumin-to-creatinine ratio (UACR) ≥ 30 mg/g persisting for ≥ 3 months, with CKD staging determined using Kidney Disease: Improving Global Outcomes (KDIGO) classifications, (3) initiation of SGLT2 inhibitor therapy or enrollment in the dose optimization program during the study period, with drugs including dapagliflozin, empagliflozin, or canagliflozin, (4) availability of at least one post-baseline eGFR measurement for longitudinal assessment, and (5) sufficient data availability in EMR and NIS systems to ascertain primary outcome measures and key covariates. Patients could be treatment-naïve to SGLT2 inhibitors or currently receiving therapy but requiring dose optimization based on clinical assessment.

#### Exclusion criteria

Patients were excluded if they met any of the following criteria: (1) diagnosis of type 1 diabetes mellitus, latent autoimmune diabetes in adults (LADA), maturity-onset diabetes of the young (MODY), or gestational diabetes mellitus, (2) acute kidney injury or reversible kidney dysfunction as the predominant kidney pathology, determined by rapid eGFR changes (> 25% within 7 days) without chronic structural changes, (3) pre-existing end-stage kidney disease (ESKD) defined as eGFR < 15 mL/min/1.73 m² or requirement for maintenance dialysis or history of kidney transplantation, (4) active glomerulonephritis requiring immunosuppressive therapy, including proliferative lupus nephritis, ANCA-associated vasculitis, or other conditions necessitating high-dose corticosteroids or cytotoxic agents, (5) concurrent participation in interventional clinical trials that could confound outcome assessment, (6) critical missing data for primary outcomes or essential covariates that could not be recovered through chart review or patient contact, and (7) life expectancy < 6 months based on terminal malignancy or end-stage organ failure documented in medical records.

#### Sample size considerations

The target sample size was 450 patients, with approximately equal distribution between structured nurse-delivered multi component group and conventional care groups. This sample size was determined based on feasibility within the single-center study period and pragmatic considerations of the retrospective design. To enhance statistical power despite the observational nature of the study, we planned to: (1) employ propensity score methods to reduce confounding and increase effective sample size, (2) utilize mixed-effects models for annualized eGFR slope estimation to maximize information from repeated measurements, and (3) conduct time-to-event analyses for composite endpoints using Cox regression with appropriate adjustment for competing risks. We report effect estimates with 95% confidence intervals to convey statistical precision and uncertainty.

### Exposure definition and group assignment

The primary exposure was enrollment in a structured, nurse delivered multi component SGLT2 inhibitor management pathway, defined by a bundled set of implementation supports including (i) more frequent scheduled contacts, (ii) protocolized laboratory monitoring and result review, and (iii) adherence reinforcement and reminder systems, in addition to standardized education and predefined escalation thresholds. Given the bundled nature of the pathway, the analysis estimates the association of the overall care delivery model with outcomes and is not designed to disentangle the independent contributions of individual components. Group assignment was determined retrospectively through review of nursing documentation, clinic attendance records, and medication dispensing patterns. Accordingly, the nurse-delivered pathway is conceptualized in this study not as a treatment exposure, but as an operational proxy for a higher-intensity, structured care delivery model incorporating frequent patient contact, protocolized monitoring, and adherence support. The analysis therefore estimates the association between care intensity and clinical outcomes, rather than the intrinsic effect of structured nurse-delivered multi component group care as an organizational form.

### Structured nurse delivered multicomponent SGLT2 management bundle and medication strategies

**Fig. 1:**
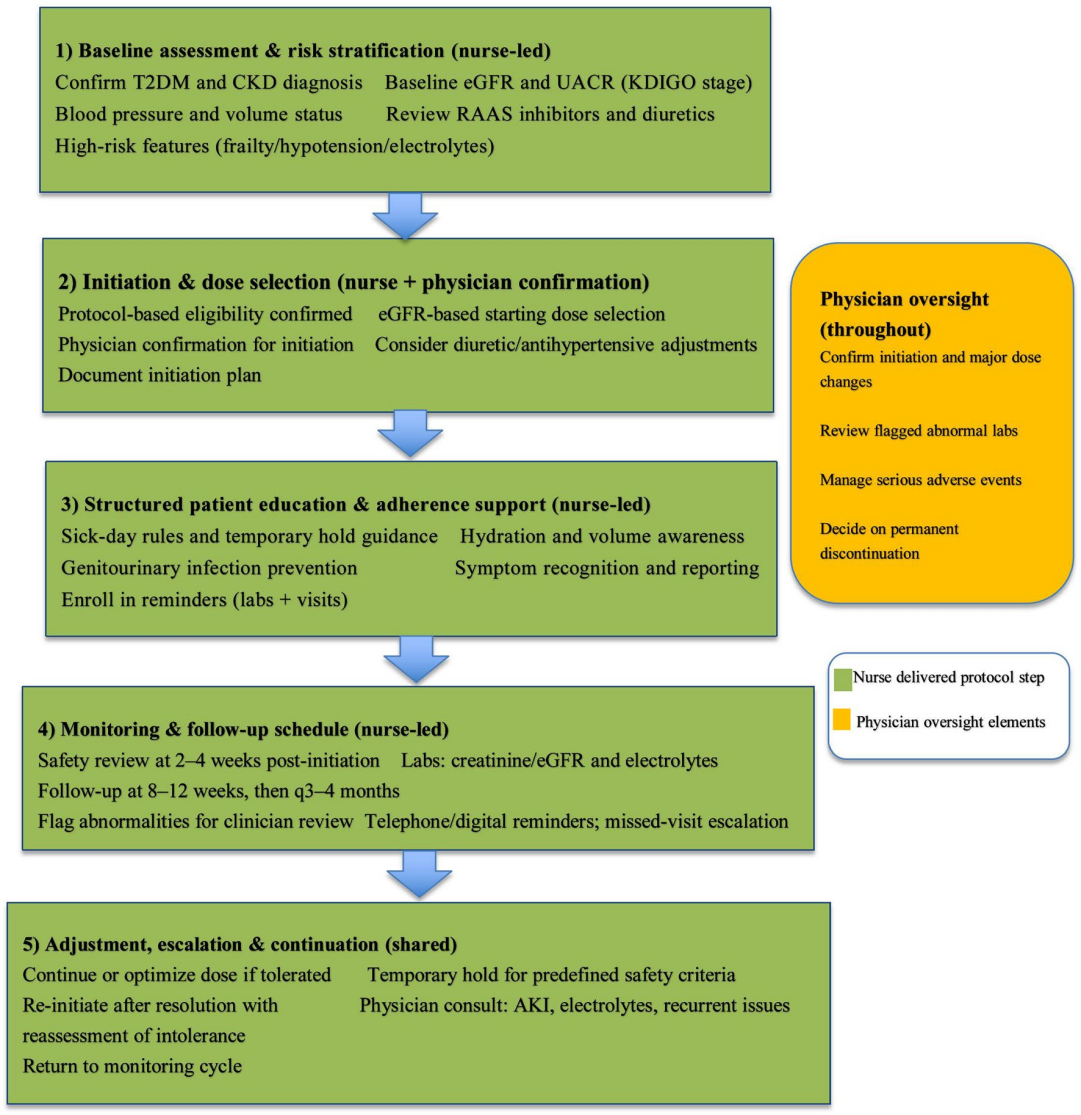
Structured nurse-delivered SGLT2 inhibitor management protocol. Schematic overview of the multi component care-delivery pathway used for SGLT2 inhibitor initiation and optimization

The structured SGLT2 inhibitor management pathway was implemented using a standardized, protocol driven care delivery model developed by a multidisciplinary team and operationalized primarily by diabetes specialist nurses under physician oversight. The protocol comprised multiple integrated components, including baseline risk assessment, eGFR-based initiation and titration, structured patient education, scheduled monitoring, and predefined escalation criteria. An overview of the protocol workflow and its core elements is provided in Fig. 1.

The structured nurse-delivered management pathway was designed as an adjunct to routine physician-led outpatient care, rather than a replacement for physician visits. Entry into the pathway occurred through physician referral, typically at the time of SGLT2 inhibitor initiation or when optimization and closer monitoring were deemed appropriate. Physicians retained primary responsibility for prescribing decisions and longitudinal disease management, while nurses delivered protocolized education, monitoring, and follow-up between scheduled physician visits.

#### Initial assessment and patient stratification

The structured nurse-delivered multi component group pathway commenced with a comprehensive baseline evaluation conducted by diabetes specialist nurses. This assessment encompassed: (1) detailed kidney function evaluation including calculation of eGFR using the CKD-EPI (Chronic Kidney Disease Epidemiology Collaboration) equation and measurement of UACR from spot urine samples, with documentation of CKD stage and albuminuria category according to KDIGO classification, (2) volume status assessment through physical examination including assessment of orthostatic blood pressure changes, peripheral edema, jugular venous pressure, and recent weight changes, along with review of current diuretic regimens and adjustment needs, (3) comprehensive metabolic and electrolyte screening including serum sodium, potassium, bicarbonate, and calculation of anion gap to detect baseline abnormalities that might influence SGLT2 inhibitor tolerability, (4) detailed medication history review with particular attention to hypoglycemia risk from concurrent antidiabetic agents, history of genitourinary infections, prior SGLT2 inhibitor use and reasons for discontinuation if applicable, and assessment of medication adherence patterns, and (5) concomitant cardiovascular medication review including RAAS inhibitors (angiotensin-converting enzyme inhibitors or angiotensin receptor blockers), loop or thiazide diuretics, and mineralocorticoid receptor antagonists, with documentation of doses and recent adjustments. Based on this comprehensive assessment, patients were stratified into risk categories: (1) low risk (eGFR ≥ 60 mL/min/1.73 m², no recent hypotension, stable electrolytes), (2) moderate risk (eGFR 30–59 mL/min/1.73 m², mild orthostatic changes, borderline electrolyte abnormalities), or (3) high risk (eGFR < 30 mL/min/1.73 m², significant volume depletion concerns, multiple electrolyte disturbances, frailty), with monitoring intensity and follow-up frequency adjusted accordingly.

#### Initiation dosing and titration algorithm

Following physician confirmation of SGLT2 inhibitor appropriateness and absence of absolute contraindications, diabetes specialist nurses implemented the initiation and titration protocol according to the following framework: (1) Initial dosing was individualized based on baseline eGFR, with standard starting doses for dapagliflozin 10 mg once daily for eGFR ≥ 25 mL/min/1.73 m², empagliflozin 10 mg once daily for eGFR ≥ 20 mL/min/1.73 m², or canagliflozin 100 mg once daily for eGFR ≥ 30 mL/min/1.73 m², in accordance with FDA-approved labeling and consideration of individual kidney function thresholds for drug initiation. (2) The titration schedule followed a structured timeline with dose assessment at 4–8 weeks after initiation, considering factors including tolerability, blood pressure response, volume status changes, electrolyte stability, and kidney function trajectory, with potential dose escalation to maximum therapeutic doses (dapagliflozin 10 mg, empagliflozin 25 mg, canagliflozin 300 mg) in patients demonstrating good tolerability and stable kidney function. (3) Coordination with concomitant medications was carefully managed, particularly with RAAS inhibitors where temporary dose reduction might be considered in patients with borderline blood pressure or significant eGFR dip (> 30% from baseline), GLP-1 receptor agonists where combination therapy was encouraged for additive cardiorenal benefits, and diuretics where dose adjustment or temporary discontinuation was considered in patients with volume depletion risk. (4) Temporary discontinuation criteria were predefined and included: volume depletion symptoms (severe orthostatic hypotension with systolic blood pressure drop > 30 mmHg, symptomatic dehydration), acute illness with potential for volume instability (gastroenteritis, acute infection with fever), planned surgical procedures with fasting requirements exceeding 24 h, acute kidney injury with rise in serum creatinine > 50% from baseline, severe genitourinary infections requiring hospitalization, or documented diabetic ketoacidosis. (5) Drug resumption after temporary discontinuation followed a standardized protocol with reassessment of volume status and kidney function, confirmation of resolution of the precipitating condition, patient re-education on preventive strategies, and consideration of dose reduction if recurrent issues occurred.

#### Patient education and adherence enhancement

A critical component of the structured nurse-delivered multi component group pathway involved intensive patient education delivered through multiple modalities. The educational intervention included: (1) Hydration counseling and sick-day management rules with explicit instructions to maintain adequate fluid intake (at least 1.5–2 L daily unless contraindicated by heart failure), recognition of dehydration symptoms including excessive thirst, dizziness, decreased urine output, and dark-colored urine, and implementation of sick-day rules mandating temporary SGLT2 inhibitor discontinuation during acute illnesses accompanied by reduced oral intake, vomiting, diarrhea, or high fever, with clear instructions on when to resume therapy. (2) Genitourinary infection prevention strategies encompassing proper perineal hygiene education, maintenance of genital area cleanliness and dryness, recognition of early symptoms of mycotic genital infections (itching, discharge, redness) and urinary tract infections (dysuria, frequency, urgency), and prompt reporting protocols for early intervention. (3) Self-monitoring and symptom reporting instructions including home blood pressure and weight monitoring for patients with heart failure or significant CKD, recognition and reporting of warning signs such as unexplained weight loss > 3 kg in one week, persistent nausea or vomiting, severe fatigue or weakness, or recurrent hypoglycemia, and provision of 24-hour hotline access to nursing staff for urgent concerns. (4) Follow-up reminder systems utilizing multiple communication channels including automated text message reminders sent 7 days and 1 day before scheduled laboratory testing, telephone calls for patients who missed appointments, We Chat or secure messaging platform follow-ups for routine check-ins, and family member notification for high-risk patients with poor prior adherence. These multi-faceted educational interventions were delivered at baseline, reinforced at each follow-up contact, and documented in the NIS to track patient understanding and engagement.

#### Laboratory monitoring and follow-up schedule

The structured nurse-delivered multi component group pathway implemented a structured monitoring schedule tailored to patient risk stratification. For standard risk patients, the schedule included: (1) Early safety assessment at 2–4 weeks post-initiation focused on electrolyte panel (sodium, potassium, bicarbonate), blood pressure measurement and orthostatic vital signs, and brief symptom review for adverse events. (2) Intermediate efficacy assessment at 8–12 weeks including comprehensive metabolic panel with eGFR calculation and serum creatinine, UACR from spot urine sample, hemoglobin A1c for glycemic control assessment, and lipid panel if clinically indicated. (3) Long-term monitoring every 12–16 weeks incorporating kidney function trending with eGFR and UACR, metabolic assessments, and comprehensive adverse event review. For high-risk patients, monitoring frequency was intensified with assessments at weeks 1–2, 4, 8, and then every 8–12 weeks, with additional monitoring triggered by specific concerns. When patients missed scheduled laboratory testing, the nursing team initiated a tiered re-engagement protocol: (1) First attempt within 48 h of missed appointment via telephone call to reschedule and address barriers, (2) Second attempt at 7 days via text message or alternative contact methods, (3) Family member notification at 14 days for patients with prior serious complications or high clinical risk, and (4) Physician notification and potential treatment reassessment for patients remaining non-adherent beyond 30 days. All monitoring results were reviewed by nursing staff within 48 h, with abnormal findings flagged using predefined thresholds (eGFR decline > 30% from baseline, potassium > 5.5 mEq/L or < 3.5 mEq/L, new or worsening albuminuria, uncontrolled blood pressure > 160/100 mmHg) for immediate physician consultation.

#### Nursing qualifications and physician oversight

The management pathway was delivered by diabetes specialist nurses with formal training in diabetes care and chronic kidney disease management and a minimum of five years of clinical experience in endocrinology or nephrology outpatient settings. These nurses were not independent advanced practice prescribers; rather, they functioned within a protocol-driven model under physician supervision. Nurses conducted structured assessments, delivered patient education, coordinated follow-up, reviewed laboratory results, and identified protocol-defined indications for dose adjustment, temporary treatment interruption, or escalation of care.

Medication initiation, permanent dose changes, and order entry were confirmed and authorized by the treating physician. Physicians also provided supervision for complex clinical decisions, including management of acute kidney injury, severe electrolyte abnormalities, recurrent intolerance, or consideration of permanent treatment discontinuation. This collaborative model ensured standardized implementation while maintaining physician accountability for prescribing and high-risk decision-making.

### Outcome measures

#### Primary outcomes

The co-primary outcomes of this study were: (1) Annualized eGFR decline rate, calculated as the change in eGFR (mL/min/1.73 m²) per year of follow-up using linear mixed-effects modeling of all available eGFR measurements, with eGFR estimated using the CKD-EPI 2021 equation incorporating serum creatinine, age, and sex, and slope estimation accounting for the characteristic acute dip in eGFR following SGLT2 inhibitor initiation (typically 2–4 weeks) followed by subsequent stabilization or attenuation of decline. (2) Composite kidney endpoint defined as the first occurrence of: sustained decline in eGFR ≥ 40% from baseline confirmed by two measurements at least 28 days apart to ensure persistency, progression to ESKD defined as eGFR < 15 mL/min/1.73 m², initiation of maintenance kidney replacement therapy (hemodialysis, peritoneal dialysis, or kidney transplantation), or death adjudicated as primarily related to kidney disease or its complications based on death certificate review and medical record documentation. The composite endpoint was analyzed as a time-to-first-event outcome using survival analysis methods, with appropriate accounting for censoring due to administrative end of follow-up, loss to follow-up, or non-kidney-related death as competing events.

#### Secondary outcomes

Secondary outcomes captured multiple dimensions of clinical effectiveness and safety: (1) UACR categorical progression or regression defined using KDIGO albuminuria stages (A1: <30 mg/g, A2: 30–299 mg/g, A3: ≥300 mg/g), with specific interest in transitions from higher to lower categories indicating beneficial effects on glomerular barrier function. (2) Medication persistence metrics including proportion of patients continuing SGLT2 inhibitor therapy at 6 and 12 months without discontinuation > 90 days, achievement of target therapeutic doses based on kidney function, and medication possession ratio calculated from pharmacy dispensing records where available. (3) Safety outcomes encompassing volume depletion-related events (emergency department visits or hospitalizations for hypotension, dehydration, or acute kidney injury attributed to volume depletion), genitourinary infections requiring antimicrobial therapy (mycotic genital infections, urinary tract infections, pyelonephritis), and diabetic ketoacidosis events, though anticipated to be rare in T2DM populations. (4) Cardiovascular and metabolic parameters including changes in systolic and diastolic blood pressure from baseline to follow-up, body weight and body mass index changes potentially reflecting favorable volume status modulation, and hemoglobin A1c trends indicating glycemic control, though not expected to show major differences given preserved beta-cell function in this CKD population. (5) Healthcare utilization patterns including frequency of unscheduled clinic visits, emergency department presentations for complications potentially related to SGLT2 inhibitor therapy or diabetic kidney disease progression, and hospitalizations for cardiovascular events, kidney disease progression, or other major morbidities during the follow-up period.

#### Implementation fidelity and care-process measures

To characterize delivery of the structured care pathway, we assessed implementation fidelity using predefined care-process indicators, including medication adherence, laboratory monitoring frequency, dose optimization rates, and nursing contact frequency. These measures reflect components of the intervention itself and were used to document pathway implementation rather than to evaluate clinical efficacy.

### Covariates and potential confounders

To account for potential confounding in this observational study, we systematically collected comprehensive baseline covariates spanning multiple domains. (1) Demographic and clinical history variables included age in years, sex, self-reported race/ethnicity, body mass index calculated from measured height and weight, smoking status (current, former, never), and duration of diabetes mellitus from diagnosis date. (2) CKD-specific characteristics encompassed baseline eGFR and CKD stage (G1-G5), baseline UACR and albuminuria category (A1-A3), presumed CKD etiology based on clinical assessment and available diagnostic workup (diabetic kidney disease, hypertensive nephrosclerosis, mixed, or other), and history of acute kidney injury episodes in the preceding 12 months. (3) Comorbidity burden was assessed through documented diagnoses of established atherosclerotic cardiovascular disease (prior myocardial infarction, coronary revascularization, stroke, or peripheral arterial disease), heart failure with preserved or reduced ejection fraction, hypertension, dyslipidemia, obesity (BMI ≥ 30 kg/m²), and other chronic conditions including chronic obstructive pulmonary disease, liver disease, and malignancy. (4) Baseline pharmacotherapy included concurrent use and doses of RAAS inhibitors (ACE inhibitors or ARBs), mineralocorticoid receptor antagonists, diuretics (loop, thiazide, thiazide-like), other antidiabetic medications (insulin, GLP-1 receptor agonists, DPP-4 inhibitors, sulfonylureas, thiazolidinediones), and cardiovascular medications (beta-blockers, calcium channel blockers, statins, antiplatelets, anticoagulants). (5) Laboratory and physiological parameters encompassed baseline hemoglobin A1c, fasting glucose, hemoglobin and hematocrit to assess anemia, serum potassium and bicarbonate for electrolyte status, systolic and diastolic blood pressure, and heart rate. (6) Healthcare access and engagement factors included insurance type (public, private, uninsured), distance from clinic, prior appointment adherence rates, and presence of designated family caregiver involvement in care. These variables were abstracted from structured EMR fields using standardized definitions, with data quality checks performed through logic rules (e.g., eGFR and serum creatinine consistency) and range restrictions. Although direct measures of health literacy and visit capability were unavailable, we incorporated several proxy indicators of healthcare access and engagement into the analysis. These included insurance type, distance from clinic, baseline outpatient visit frequency in the 12 months preceding SGLT2 inhibitor initiation, prior laboratory testing adherence, and documented involvement of family caregivers. These variables were selected a priori to partially capture differences in healthcare accessibility and capacity to engage in longitudinal follow-up.

### Data sources, extraction, and quality control

Data were systematically extracted from multiple electronic sources within the hospital information system. The EMR provided clinical documentation including physician notes, laboratory results, vital signs, and medication orders. The NIS captured nursing-specific documentation including pathway enrollment status, patient education sessions, telephone follow-up encounters, and adverse event reports. The pharmacy system supplied medication dispensing records including fill dates, quantities, and days’ supply for adherence calculations. Data extraction was performed using structured query language (SQL) queries developed collaboratively by clinical investigators and health informatics specialists, with queries validated against manual chart review for a random sample of 50 patients to ensure accuracy. Quality control procedures included: (1) Dual independent data extraction by two trained research coordinators for key outcome variables and covariates, with discrepancies resolved through consensus review with a third investigator. (2) Logic checks for internal consistency including verification that eGFR calculations matched recorded serum creatinine values using the CKD-EPI equation, confirmation that medication start dates preceded outcome event dates, and validation that vital signs fell within physiologically plausible ranges. (3) Outlier detection through visualization of continuous variable distributions and investigation of extreme values through source document review to distinguish true values from data entry errors. (4) Missing data patterns were characterized, including calculation of missingness percentages for each variable, assessment of relationships between missingness and patient characteristics to evaluate whether data were missing completely at random (MCAR), missing at random (MAR), or missing not at random (MNAR), and documentation of reasons for missingness where identifiable from chart review. For critical variables with > 20% missingness that could not be recovered, sensitivity analyses assessed potential impact on study conclusions.

### Statistical analysis

#### Descriptive statistics and group comparisons

Baseline characteristics were summarized using appropriate descriptive statistics. Continuous variables were presented as mean ± standard deviation (SD) for approximately normally distributed variables or median (interquartile range, IQR) for skewed distributions, with normality assessed using histograms, Q-Q plots, and Shapiro-Wilk tests. Categorical variables were reported as frequencies and percentages. Unadjusted comparisons between structured nurse-delivered multi component group and conventional care groups employed independent samples t-tests for normally distributed continuous variables, Mann-Whitney U tests for non-normally distributed continuous variables, and chi-square tests or Fisher’s exact tests for categorical variables, with statistical significance defined as two-sided *P* < 0.05. Effect sizes were calculated to assess clinical meaningfulness of differences, including standardized mean differences (Cohen’s d) for continuous variables and risk ratios or odds ratios for categorical variables.

#### Propensity score methods and covariate balance

To address confounding by indication and selection bias inherent in observational comparisons, we employed propensity score methodology. The propensity score, defined as the conditional probability of receiving structured nurse-delivered multi component group management given observed baseline covariates, was estimated using multivariable logistic regression. The propensity score model included all clinically relevant covariates hypothesized to influence both treatment assignment and outcomes, specifically: age, sex, baseline eGFR, baseline UACR, CKD stage, presence of cardiovascular disease, heart failure, diabetes duration, hemoglobin A1c, systolic blood pressure, body mass index, use of RAAS inhibitors, use of diuretics, smoking status, and comorbidity count. We evaluated alternative weighting schemes including inverse probability of treatment weighting (IPTW) using stabilized weights and overlap weighting to optimize covariate balance while maintaining effective sample size. Covariate balance after propensity score application was assessed using standardized mean differences (SMD), with SMD < 0.10 considered indicative of adequate balance. We examined propensity score distributions across treatment groups using density plots and evaluated common support assumption by trimming extreme propensity score values (beyond 0.05 and 0.95) if necessary. Sensitivity to propensity score model specification was evaluated by comparing results across different covariate inclusion strategies. The propensity score model included demographic, clinical, and healthcare engagement variables hypothesized to influence both pathway enrollment and outcomes. In addition to traditional clinical covariates, we incorporated proxy measures of visit capability and healthcare access, including insurance type, distance from clinic, baseline outpatient visit frequency in the preceding 12 months, and prior laboratory adherence. These additions were intended to mitigate confounding related to differential ability to engage in frequent follow-up and monitoring within the structured care pathway.

#### Primary outcome modeling

For the annualized eGFR decline rate, we implemented linear mixed-effects models that account for within-patient correlation of repeated eGFR measurements over time. The model included fixed effects for time (continuous), treatment group (structured nurse-delivered multi component group vs. conventional), and their interaction term, with the interaction coefficient representing the difference in eGFR slopes between groups. Random effects for both intercept and slope were specified to capture between-patient heterogeneity in baseline eGFR and rate of decline. The model was adjusted for propensity score quintiles or incorporated propensity score weights to control for confounding. To appropriately handle the acute eGFR dip following SGLT2 inhibitor initiation, we specified a piece wise linear spline with a knot at 4 weeks post-initiation, allowing separate slope estimation for the acute and chronic phases. This approach aligns with established patterns of SGLT2 inhibitor-induced eGFR changes observed in clinical trials [18, 19]. For the composite kidney endpoint, we employed Cox proportional hazards regression to estimate hazard ratios (HR) and 95% confidence intervals (CI) comparing structured nurse-delivered multi component group versus conventional management. The Cox model was stratified by propensity score quintiles or incorporated propensity score weights. The proportional hazards assumption was evaluated using Schoenfeld residuals and log-log plots. Given the potential for non-kidney-related death as a competing risk, we conducted sensitivity analyses using Fine-Gray subdistribution hazard models to assess robustness of conclusions. Time-to-event was measured from the date of SGLT2 inhibitor initiation or pathway enrollment until the first occurrence of any composite endpoint component, with censoring at loss to follow-up, administrative end of study, or competing events.

#### Sensitivity analyses for informative observation bias

Because laboratory monitoring frequency differed by group and was an explicit component of the structured care pathway, estimation of longitudinal eGFR slopes may be susceptible to informative observation (visit) bias. To characterize the observation process, we summarized the timing of eGFR measurements relative to baseline and visualized these distributions using density and raincloud plots stratified by study group (Supplementary Figure S1). These visualizations demonstrate higher measurement intensity and earlier follow-up in the structured pathway group, consistent with protocolized monitoring. Accordingly, longitudinal slope estimates should be interpreted as reflecting both pharmacologic effects and differences in care intensity, and residual informative observation bias cannot be fully excluded in this retrospective analysis.

#### Secondary outcomes and subgroup analyses

For UACR categorical transitions, we utilized ordinal logistic regression models (proportional odds models) to assess shifts across albuminuria categories, with adjustment for propensity score and baseline UACR category. Safety outcomes including adverse events were analyzed using Poisson or negative binomial regression models with offset terms for person-time to estimate incidence rate ratios, accounting for differential follow-up duration across patients. Subgroup analyses were pre specified to evaluate treatment effect heterogeneity across clinically relevant patient strata: (1) baseline kidney function (eGFR ≥ 45 vs. <45 mL/min/1.73 m²), (2) baseline albuminuria severity (UACR < 300 vs. ≥300 mg/g), (3) age group (≥ 65 vs. <65 years), and (4) presence versus absence of heart failure at baseline. Subgroup-specific treatment effects were estimated by including interaction terms between treatment group and subgroup indicators in regression models, with interaction P-values < 0.10 considered suggestive of effect modification. Forest plots were constructed to visualize treatment effects across subgroups with 95% confidence intervals, facilitating assessment of consistency versus heterogeneity of effects.

#### Missing data handling and sensitivity analyses

Missing covariate and outcome data were addressed using multiple imputation by chained equations (MICE) with 20 imputed datasets generated to ensure stable point estimates and confidence intervals. The imputation model included all variables in the analysis model plus auxiliary variables correlated with missingness or outcome to satisfy the MAR assumption. Imputation diagnostics included trace plots to assess convergence and comparison of imputed versus observed value distributions. Primary analyses were conducted across all imputed datasets with results combined using Rubin’s rules. To assess robustness of findings, we conducted comprehensive sensitivity analyses: (1) complete case analysis restricted to patients with no missing data for primary outcome or key covariates to evaluate potential bias from imputation, (2) alternative censoring assumptions for patients lost to follow-up, including best-case (no events) and worst-case (events immediately after loss) scenarios, (3) varying exposure definitions by altering the threshold for pathway disengagement from 90 to 60 or 120 days, (4) excluding patients with very short follow-up (< 3 months) who contributed limited longitudinal information, and (5) adjustment for time-varying covariates including changes in concurrent medications during follow-up using time-dependent Cox models. Consistency of findings across sensitivity analyses strengthened confidence in primary results.

#### Multiple comparisons and statistical software

Given the multiple secondary outcomes and subgroup analyses, we controlled the false discovery rate (FDR) using the Benjamini-Hochberg procedure to account for multiplicity, with FDR-adjusted P-values reported alongside unadjusted P-values. All hypothesis tests were two-sided with significance level α = 0.05 before multiplicity adjustment. Statistical analyses were conducted using R version 4.3.1 (R Foundation for Statistical Computing, Vienna, Austria) with key packages including lme4 for mixed-effects models, survival and cmprsk for time-to-event analyses, MatchIt for propensity score implementation, and mice for multiple imputation. SAS version 9.4 (SAS Institute Inc., Cary, NC) was used for sensitivity analyses to confirm consistency of results across statistical platforms.

### Bias mitigation strategies

Baseline characteristics were summarized using mean ± standard deviation for normally distributed continuous variables, median (inter quartile range) for skewed distributions, and frequencies with percentages for categorical variables. Unadjusted group comparisons employed t-tests, Mann-Whitney U tests, or chi-square tests as appropriate, with two-sided *P* < 0.05 considered statistically significant. To address confounding in this observational study, we employed propensity score methodology. Propensity scores were estimated using multivariable logistic regression including age, sex, baseline eGFR, baseline UACR, CKD stage, cardiovascular disease, heart failure, diabetes duration, hemoglobin A1c, systolic blood pressure, body mass index, RAAS inhibitor use, diuretic use, smoking status, and comorbidity count. Inverse probability of treatment weighting with stabilized weights was applied to balance baseline covariates, with standardized mean differences < 0.10 indicating adequate balance. For the annualized eGFR decline rate, we used linear mixed-effects models with random intercepts and slopes, including fixed effects for time, treatment group, and their interaction. A piecewise linear spline with knot at 4 weeks post-initiation accounted for the acute eGFR dip following SGLT2 inhibitor initiation. For the composite kidney endpoint, Cox proportional hazards regression stratified by propensity score quintiles estimated hazard ratios and 95% confidence intervals. The proportional hazards assumption was evaluated using Schoenfeld residuals. Fine-Gray subdistribution hazard models addressed competing risks from non-kidney-related death. Secondary outcomes were analyzed using ordinal logistic regression for UACR transitions and Poisson or negative binomial regression for adverse events. Prespecified subgroup analyses evaluated treatment heterogeneity by baseline eGFR (≥ 45 vs. <45 mL/min/1.73 m²), UACR (< 300 vs. ≥300 mg/g), age (≥ 65 vs. <65 years), and heart failure status, with interaction P-values < 0.10 considered suggestive of effect modification. Missing data were handled using multiple imputation by chained equations with 20 imputed datasets, with results combined using Rubin’s rules. Sensitivity analyses included complete case analysis, alternative censoring assumptions, varying exposure definitions, and time-varying covariate adjustment. The Benjamini-Hochberg procedure controlled false discovery rate for multiple testing. Statistical analyses were conducted using R version 4.3.1 with packages including lme4, survival, cmprsk, MatchIt, and mice, with SAS version 9.4 used for confirmatory sensitivity analyses.

## Results

### Study population and baseline characteristics

**Fig. 2:**
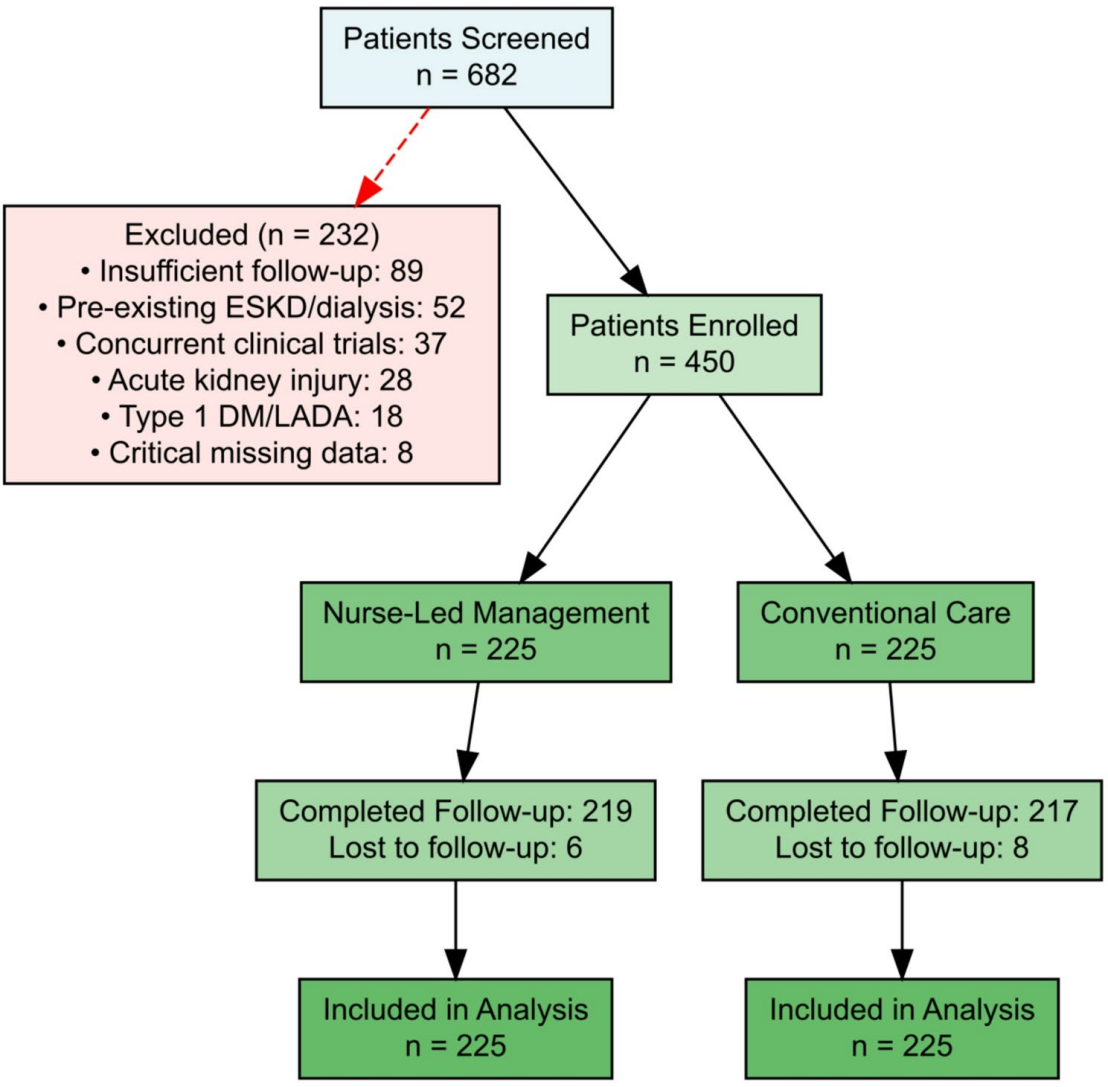
Study flow diagram. Flow diagram of patient selection and cohort assembly. A total of 682 patients with type 2 diabetes mellitus (T2DM) and chronic kidney disease (CKD) were screened between February 2024 and February 2025. After application of inclusion and exclusion criteria, 450 patients were included in the final analysis, with 225 assigned to the structured nurse-delivered management pathway and 225 to conventional care. Reasons for exclusion are shown. Abbreviations: CKD, chronic kidney disease; EMR, electronic medical record

Between February 2024 and February 2025, a total of 682 patients with T2DM and CKD were screened for eligibility. After applying inclusion and exclusion criteria, 450 patients were included in the final analysis cohort, with 225 patients in the structured nurse-delivered multi component group and 225 patients in the conventional care group. Patient flow through the study is depicted in Fig. 2. The baseline characteristics of the study population are presented in Table 1. Prior to propensity score adjustment, there were some imbalances between groups, with the conventional care group having slightly older age (mean 62.4 ± 9.8 vs. 60.5 ± 10.2 years), lower baseline eGFR (median 38.5 vs. 41.2 mL/min/1.73 m²), and higher prevalence of heart failure (32.4% vs. 24.0%). After application of inverse probability of treatment weighting using propensity scores, excellent balance was achieved across all measured covariates, with all standardized mean differences below 0.10 (Table 2). The weighted cohort had a mean age of 61.5 years, 58.2% male sex, median diabetes duration of 12 years, mean baseline eGFR of 40.1 mL/min/1.73 m², and median UACR of 285 mg/g. The majority of patients (87.3%) were receiving RAAS inhibitor therapy at baseline.

**Table 1:** Baseline characteristics of study participants (Unweighted)

Variable	Overall (*n* = 450)	Structured nurse group (*n* = 225)	Conventional (*n* = 225)	SMD
Age (years), mean ± SD	61.5 ± 10.1	60.5 ± 10.2	62.4 ± 9.8	0.19
Male sex, n (%)	262 (58.2)	128 (56.9)	134 (59.6)	0.05
Diabetes duration (years), median(IQR)	12 (8–17)	11 (7–16)	13 (9–18)	0.15
BMI (kg/m²), mean ± SD	28.6 ± 4.7	28.4 ± 4.5	28.8 ± 4.9	0.08
eGFR (mL/min/1.73 m²), median (IQR)	40.1 (32.5–51.8)	41.2 (33.8–52.6)	38.5 (31.2–50.3)	0.16
UACR (mg/g), median (IQR)	285 (120–680)	265(115–625)	310 (135–740)	0.12
**CKD Stage**,** n (%)**				
G2 (eGFR 60–89)	78 (17.3)	42 (18.7)	36 (16.0)	0.07
G3a (eGFR 45–59)	142 (31.6)	74 (32.9)	68 (30.2)	0.06
G3b (eGFR 30–44)	168 (37.3)	82 (36.4)	86 (38.2)	0.04
G4 (eGFR 15–29)	62 (13.8)	27 (12.0)	35 (15.6)	0.11
**Albuminuria Category**,** n (%)**				
A2 (30–299 mg/g)	246 (54.7)	128 (56.9)	118 (52.4)	0.09
A3 (≥ 300 mg/g)	204 (45.3)	97 (43.1)	107 (47.6)	0.09
Heart failure, n (%)	127 (28.2)	54 (24.0)	73 (32.4)	0.19
Cardiovascular disease, n (%)	142 (31.6)	68 (30.2)	74 (32.9)	0.06
Hypertension, n (%)	428 (95.1)	213 (94.7)	215 (95.6)	0.04
RAAS inhibitor use, n (%)	393 (87.3)	195 (86.7)	198 (88.0)	0.04
Diuretic use, n (%)	178 (39.6)	84 (37.3)	94 (41.8)	0.09
HbA1c (%), mean ± SD	8.2 ± 1.4	8.1 ± 1.3	8.3 ± 1.5	0.14
Systolic BP (mmHg), mean ± SD	138.4 ± 16.2	137.2 ± 15.8	139.6 ± 16.6	0.15

Note: BMI, body mass index; BP, blood pressure; CKD, chronic kidney disease; eGFR, estimated glomerular filtration rate; HbA1c, hemoglobin A1c; IQR, interquartile range; RAAS, renin-angiotensin-aldosterone system; SD, standard deviation; SMD, standardized mean difference; UACR, urinary albumin-to-creatinine ratio

**Table 2:** Covariate balance before and after propensity score weighting

Variable	SMD Before Weighting	SMD After Weighting
Age	0.19	0.03
Male sex	0.05	0.02
Diabetes duration	0.15	0.04
BMI	0.08	0.03
Baseline eGFR	0.16	0.05
Baseline UACR	0.12	0.04
Heart failure	0.19	0.06
Cardiovascular disease	0.06	0.03
RAAS inhibitor use	0.04	0.02
Diuretic use	0.09	0.03
HbA1c	0.14	0.04
Systolic BP	0.15	0.05

Note: All SMD values after weighting are < 0.10, indicating adequate balance. BMI, body mass index; BP, blood pressure; eGFR, estimated glomerular filtration rate; HbA1c, hemoglobin A1c; RAAS, renin-angiotensin-aldosterone system; SMD, standardized mean difference; UACR, urinary albumin-to-creatinine ratio

### Pathway implementation and adherence

**Fig. 3:**
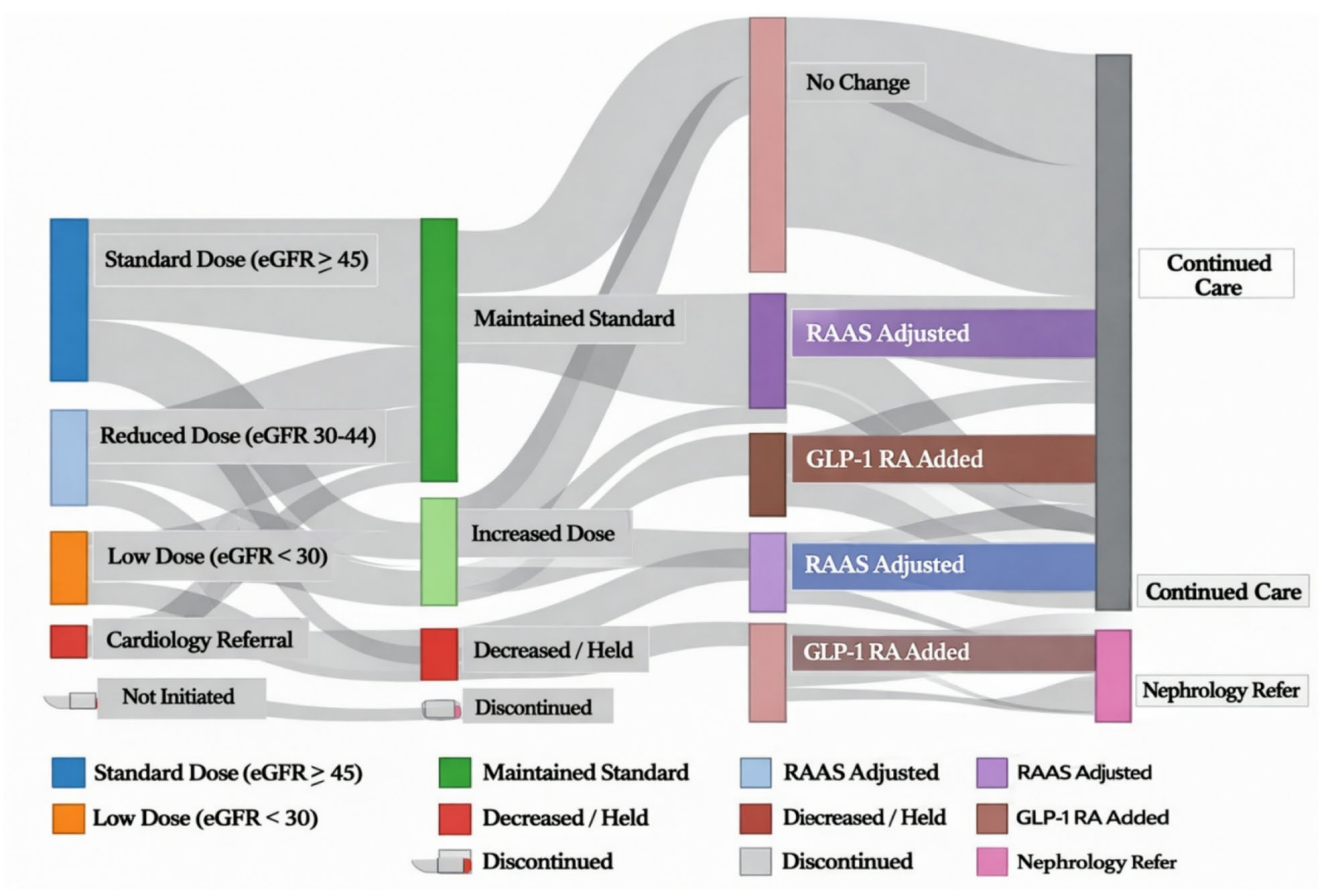
Sankey diagram of SGLT2 inhibitor dose trajectories. Sankey diagram illustrating SGLT2 inhibitor initiation, dose adjustment, and discontinuation pathways over the first 12 weeks of follow-up. Flow widths represent the proportion of patients transitioning between dosing states. Blue streams indicate patients managed within the structured nurse-delivered pathway, while gray streams represent conventional care. Arrows depict dose escalation, maintenance, temporary interruption, or discontinuation. Abbreviations: SGLT2, sodium–glucose cotransporter-2; eGFR, estimated glomerular filtration rate

Implementation metrics demonstrated higher engagement and adherence in the structured nurse-delivered multi component group compared to conventional care (Table 3). The structured nurse-delivered multi component group achieved significantly higher rates of appropriate initial dosing (91.6% vs. 78.2%, absolute difference 13.4%, 95% CI 6.8–20.0%, *P* < 0.001), with doses aligned with kidney function-based recommendations. Dose optimization completion within 8–12 weeks was markedly better with structured nurse-delivered multi component group management (85.3% vs. 58.7%, *P* < 0.001), indicating more systematic titration practices. Laboratory monitoring compliance at 12 weeks was substantially higher in the structured nurse-delivered multi component group (82.3% vs. 64.7%, *P* < 0.001), reflecting the effectiveness of reminder systems and follow-up protocols. Patient-reported medication adherence assessed through validated questionnaires showed fewer patients with self-reported missed or incorrect doses in the structured nurse-delivered multi component group (11.6% vs. 23.1%, *P* = 0.002). Temporary drug discontinuation due to adverse events occurred less frequently with structured nurse-delivered multi component group management (8.4% vs. 15.2%, *P* = 0.012), suggesting more effective patient education regarding sick-day rules and preventive strategies. Figure 3 illustrates the medication adjustment pathways using a Sankey diagram, demonstrating more structured dose titration and optimization patterns in the structured nurse-delivered multi component group compared to the variable trajectories observed with conventional care.

**Table 3:** Implementation metrics and adherence indicators

Indicator	Structured nurse-group(*n* = 225)	Conventional (*n* = 225)	Difference (95% CI)	*P* Value
Appropriate initial dose, %	91.6	78.2	13.4 (6.8–20.0)	< 0.001
Dose optimization completed (8–12 wks), %	85.3	58.7	26.6 (18.4–34.8)	< 0.001
Laboratory monitoring at 12 weeks, %	82.3	64.7	17.6 (9.5–25.7)	< 0.001
Self-reported adherence issues, %	11.6	23.1	-11.5 (-18.9 to -4.1)	0.002
Adverse event-related discontinuation, %	8.4	15.2	-6.8 (-12.6 to -1.0)	0.012
Medication persistence at 6 months, %	88.4	76.9	11.5 (4.2–18.8)	0.001
Nursing contact frequency (per 6 months), median (IQR)	5 (4–7)	1 (0–2)	—	< 0.001

Note: CI, confidence interval; IQR, interquartile range

### Implementation fidelity and care-process indicators

Care-process indicators demonstrated higher implementation fidelity in the structured pathway group, including more frequent laboratory monitoring, higher rates of dose optimization, and greater patient contact. These findings document successful delivery of the intervention as designed and provide contextual information for interpretation of downstream clinical outcomes (Table 4). After propensity score weighting and adjustment for residual confounding, the structured nurse-delivered multi component group demonstrated a slower annualized eGFR decline rate compared to conventional care (estimated difference in slope 1.28 mL/min/1.73 m²/year, 95% CI 0.65–1.91, *P* < 0.001), indicating preserved kidney function over time. This effect was evident beyond the initial acute eGFR dip period, with the chronic phase slope showing sustained attenuation of decline. For the composite kidney endpoint, 32 events occurred in the structured nurse-delivered multi component group compared to 56 events in the conventional care group during follow-up. The adjusted hazard ratio was 0.68 (95% CI 0.52–0.89, *P* = 0.004), representing a 32% relative risk reduction with structured nurse-delivered multi component group management. Competing risk analysis using Fine-Gray models yielded similar results (sub distribution HR 0.71, 95% CI 0.54–0.92), confirming robustness to different analytical approaches.

**Table 4:** Implementation fidelity and Care-Process indicators

Outcome		Structured nurse-group	Conventional	Effect Estimate (95% CI)	*P* Value
**Clinical Outcomes**					
eGFR slope (mL/min/1.73 m²/year), mean ± SE		-2.85 ± 0.28	-4.13 ± 0.31	Diff: 1.28 (0.65–1.91)	< 0.001
Composite kidney endpoint, events/100 person-years		7.2	10.8	HR: 0.68 (0.52–0.89)	0.004
**Secondary Outcomes**					
UACR category improvement, %		28.4	16.7	OR: 2.03 (1.31–3.15)	0.001
Medication persistence at 12 months, %		84.2	71.3	RR: 1.18 (1.08–1.29)	< 0.001
Genitourinary infections, events/100 person-years		5.8	6.5	IRR: 0.89 (0.54–1.47)	0.65
Volume depletion events, events/100 person-years		2.1	3.6	IRR: 0.58 (0.31–1.09)	0.088
Diabetic ketoacidosis, n		0	0	—	—
Change in systolic BP (mmHg), mean ± SD		-5.2 ± 12.4	-3.1 ± 13.8	Diff: -2.1 (-4.8 to 0.6)	0.082
Change in body weight (kg), mean ± SD		-2.3 ± 4.1	-1.9 ± 4.5	Diff: -0.4 (-1.2 to 0.4)	0.31
Change in HbA1c (%), mean ± SD		-0.6 ± 1.1	-0.5 ± 1.2	Diff: -0.1 (-0.3 to 0.1)	0.38

Note: BP, blood pressure; CI, confidence interval; Diff, difference; eGFR, estimated glomerular filtration rate; HbA1c, hemoglobin A1c; HR, hazard ratio; IRR, incidence rate ratio; OR, odds ratio; RR, risk ratio; SD, standard deviation; SE, standard error; UACR, urinary albumin-to-creatinine ratio. These variables represent intervention components and care-process indicators and are not interpreted as measures of clinical efficacy

**Fig. 4:**
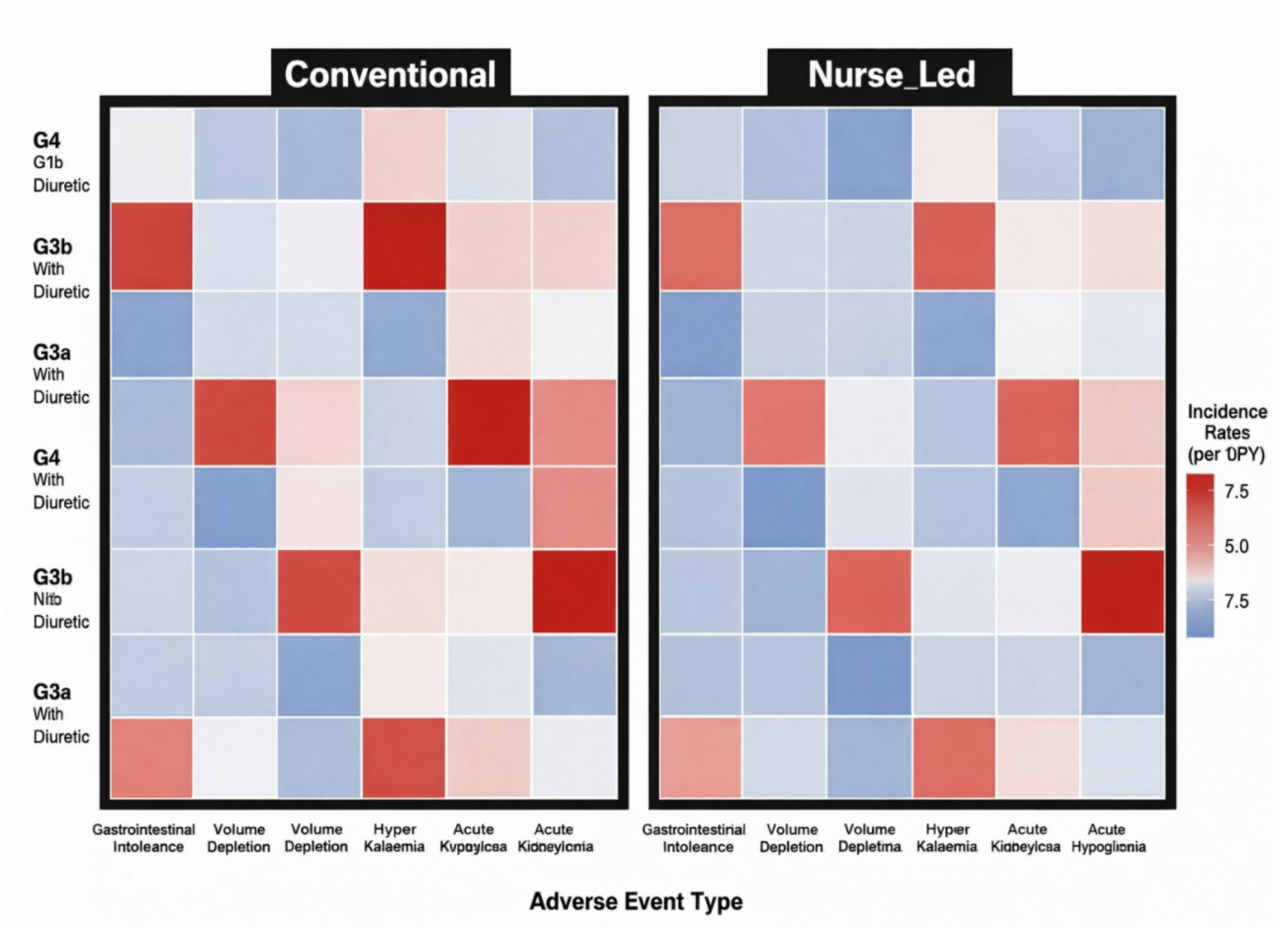
Heatmap of safety events. Heatmap displaying incidence rates of safety events stratified by baseline CKD stage and diuretic use. Color intensity corresponds to event frequency, with darker shades indicating higher incidence. Safety events include volume depletion related encounters and genitourinary infections requiring treatment. Rows represent CKD stages (G2–G4), and columns indicate diuretic exposure status. Abbreviations: CKD, chronic kidney disease

**Fig. 5:**
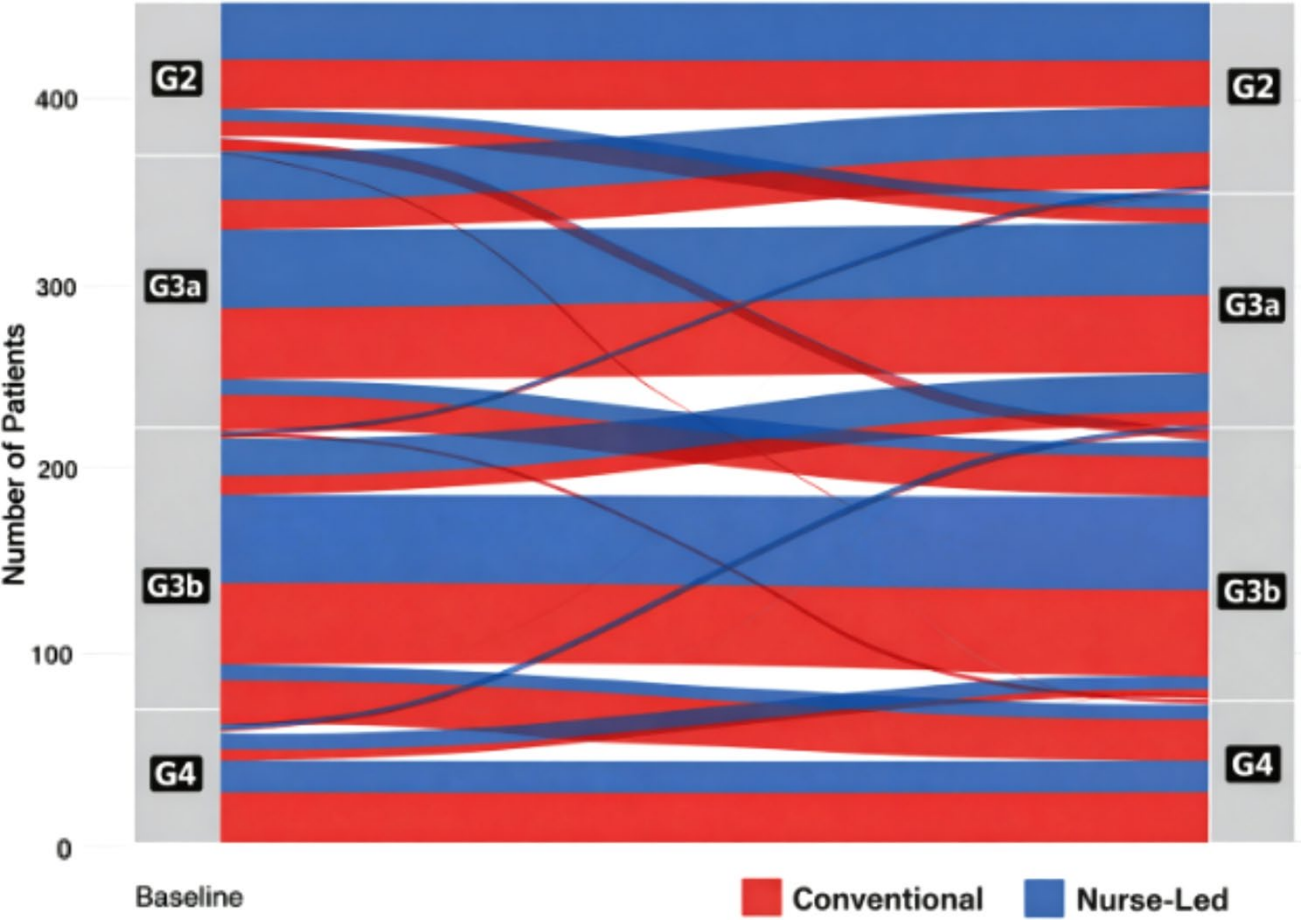
Alluvial plot of CKD stage transitions. Alluvial diagram illustrating transitions in CKD stage from baseline to last follow-up. Stream widths reflect the proportion of patients within each CKD stage over time. Blue flows represent patients in the structured nurse-delivered pathway, and gray flows represent conventional care. Stability or improvement in CKD stage is visually contrasted with progression. Abbreviations: CKD, chronic kidney disease; eGFR, estimated glomerular filtration rate

Among secondary outcomes, the structured nurse-delivered multi component group showed more favorable albuminuria category transitions, with 28.4% of patients improving from A3 to A2/A1 compared to 16.7% in conventional care (OR 2.03, 95% CI 1.31–3.15, *P* = 0.001). Safety outcomes demonstrated reassuring profiles in both groups. Genitourinary infections requiring treatment occurred at similar rates (incidence rate ratio 0.89, 95% CI 0.54–1.47, *P* = 0.65), suggesting comparable infectious complication risks. Volume depletion-related healthcare encounters were numerically lower with structured nurse-delivered multi component group management (incidence rate ratio 0.58, 95% CI 0.31–1.09, *P* = 0.088), though not reaching statistical significance. No cases of diabetic ketoacidosis occurred in either group, consistent with the low risk in T2DM populations. Blood pressure reduction from baseline was slightly greater in the structured nurse-delivered multi component group (systolic BP change − 5.2 ± 12.4 vs. -3.1 ± 13.8 mmHg, *P* = 0.082), while body weight changes were similar between groups. Hemoglobin A1c decreased modestly in both groups without significant between-group differences, as expected given that glycemic improvement was not the primary focus of this CKD-targeted intervention. Figure 4 presents a heatmap of safety event rates stratified by CKD stage and diuretic use, demonstrating that adverse events remained infrequent across risk strata with no major safety signals identified. Figure 5 displays CKD stage transitions using an alluvial diagram, illustrating greater stability or improvement in kidney function category with structured nurse-delivered multi component group management compared to progression observed more frequently with conventional care.

In sensitivity analyses incorporating proxy measures of healthcare access and engagement into the propensity score model, treatment effect estimates for both the annualized eGFR slope and the composite kidney endpoint were materially unchanged. Quantitative bias analysis using E-values indicated that an unmeasured confounder would need to be associated with both pathway enrollment and the composite kidney endpoint by a risk ratio of at least 2.30 to fully explain the observed hazard ratio of 0.68. The corresponding E-value for the upper bound of the 95% confidence interval (HR 0.89) was 1.49, suggesting moderate robustness of the findings to unmeasured confounding.

As expected, the structured pathway group underwent more frequent laboratory monitoring, with earlier post-initiation assessments and a higher density of eGFR measurements over follow-up, as illustrated in Supplementary Fig. 1. These patterns reflect the protocolized monitoring embedded within the structured care pathway and underscore that observed differences in eGFR trajectories should be interpreted in the context of greater care intensity rather than visit-independent treatment effects.

**Fig. 6:**
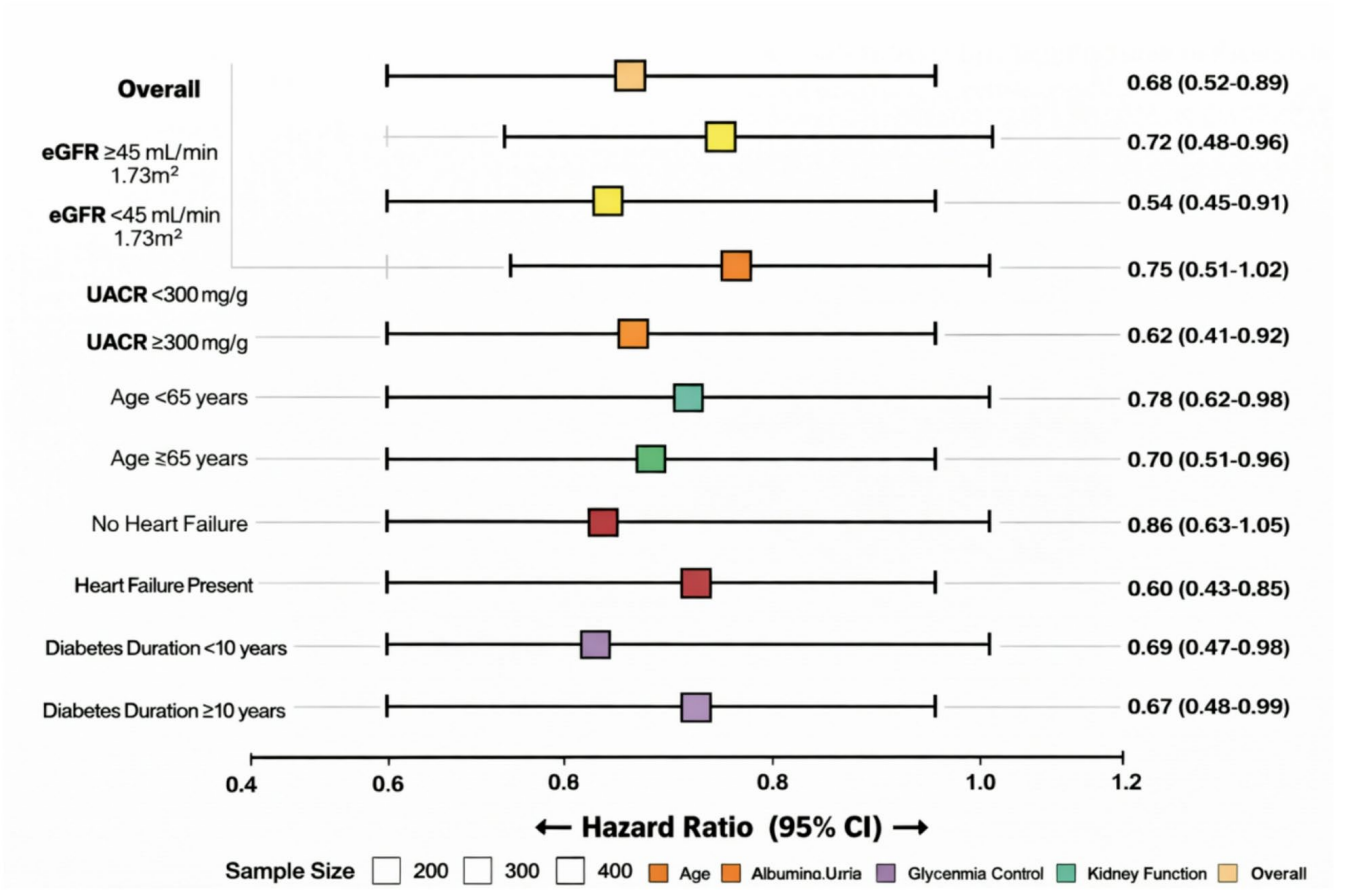
Subgroup analysis forest plot. Forest plot showing hazard ratios (HRs) and 95% confidence intervals for the composite kidney endpoint across prespecified subgroups. Squares indicate point estimates, with horizontal lines representing 95% confidence intervals. The vertical dashed line denotes the null effect (HR = 1.0). Subgroups include baseline eGFR category, albuminuria severity, age group, and heart failure status. Abbreviations: HR, hazard ratio; eGFR, estimated glomerular filtration rate; UACR, urinary albumin-to-creatinine ratio

**Fig. 7:**
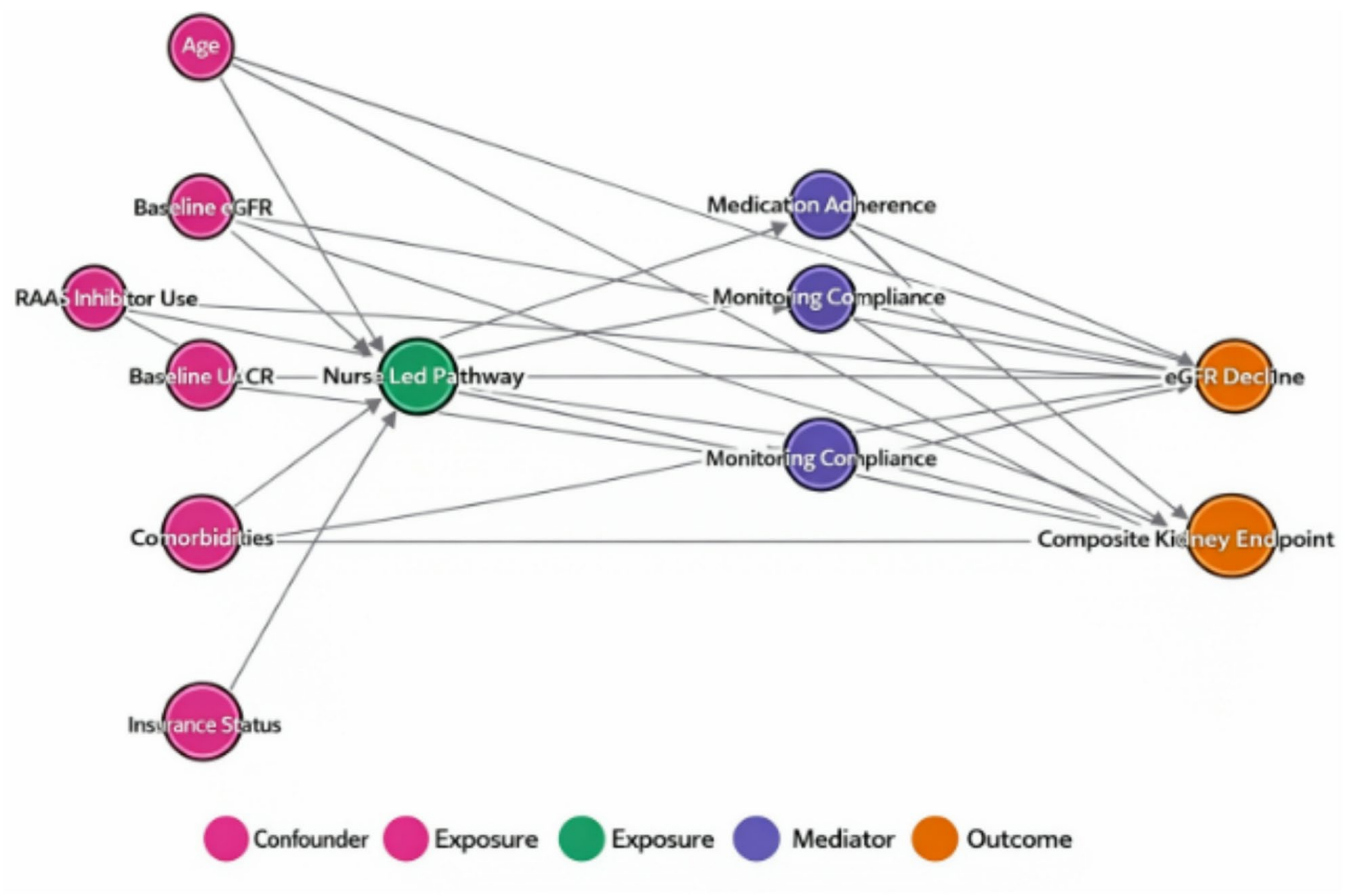
Directed acyclic graph (DAG). Directed acyclic graph illustrating hypothesized causal relationships between pathway participation, measured covariates, unmeasured confounders, and kidney outcomes. Solid arrows represent assumed causal pathways, while dashed arrows indicate potential unmeasured confounding related to patient motivation, health literacy, and access to care. The DAG informed covariate selection for propensity score modeling

Pre specified subgroup analyses demonstrated consistency of the treatment effect across clinically relevant patient strata (Fig. 6). The beneficial effect on the composite kidney endpoint was observed across all subgroups including patients with more advanced kidney disease (eGFR < 45 mL/min/1.73 m², HR 0.64, 95% CI 0.45–0.91) and those with higher albuminuria (UACR ≥ 300 mg/g, HR 0.62, 95% CI 0.42–0.92), with no significant interaction detected (P for interaction = 0.72 and 0.58, respectively). Similarly, age and heart failure status did not significantly modify treatment effects, suggesting broad applicability of the structured nurse-delivered multi component group pathway across diverse CKD populations. Sensitivity analyses supported the robustness of primary findings, with consistent results observed in complete case analysis, alternative censoring assumptions, and varying exposure definitions. Figure 7 presents a directed acyclic graph (DAG) illustrating the hypothesized causal relationships between structured nurse-delivered multi component group management, confounding variables, and clinical outcomes, which guided the selection of adjustment variables in statistical models.

## Discussion

This real-world retrospective study found that participation in a structured, nurse delivered SGLT2 inhibitor initiation and optimization pathway was associated with attenuated kidney function decline and a lower risk of adverse kidney outcomes in patients with T2DM and CKD. The observed associations occurred in the context of a multi component care delivery model characterized by more frequent patient contact, protocolized monitoring, adherence reinforcement, and standardized education. These findings suggest that implementation barriers limiting the translation of SGLT2 inhibitor trial efficacy into routine clinical practice may be partially addressed through structured care delivery strategies, rather than through pharmacologic therapy alone. Importantly, the observed associations should be interpreted as reflecting the effects of enhanced care intensity and structured implementation support, rather than evidence of intrinsic superiority of the structured nurse-delivered multi component group organizational model itself. Measures such as adherence, monitoring frequency, and dose optimization represent intervention components and indicators of implementation fidelity; they are not clinical outcomes and should not be interpreted as evidence of treatment efficacy.

The magnitude of benefit observed in our study, while somewhat smaller than the landmark DAPA-CKD and EMPA-KIDNEY trials which reported 39% and 28% relative risk reductions respectively, nonetheless demonstrates meaningful clinical impact in a real-world setting with broader patient eligibility and usual-care background therapy [5, 20]. The attenuated eGFR decline rate difference of 1.28 mL/min/1.73 m²/year between structured nurse-delivered multi component group and conventional management groups translates to preservation of approximately 6.4 mL/min/1.73 m² of kidney function over 5 years, potentially delaying progression to end-stage kidney disease by several years in at-risk populations. This effect size is clinically significant and aligns with observations from observational SGLT2 inhibitor studies in diabetes populations [18, 21].

The mechanisms underlying the observed associations likely extend beyond the pharmacological effects of SGLT2 inhibitors alone and reflect features of the broader care delivery bundle. Our findings align with broader literature demonstrating that structured nurse-delivered multi component group interventions improve chronic disease management through enhanced patient engagement, structured follow-up protocols, and systematic implementation of evidence-based care algorithms [14, 16, 22, 23]. Several specific pathway components may have contributed to observed benefits. First, standardized initiation protocols with individualized dosing based on kidney function and volume status assessment likely reduced inappropriate underdosing or hesitancy to initiate therapy that may occur in conventional care settings where providers are less familiar with these agents in CKD populations. The uptake barriers related to uncertainty and lack of familiarity among healthcare professionals can be partially mitigated through protocol-driven nursing implementation [8].

Second, intensive patient education regarding sick-day rules, hydration, and symptom recognition empowered patients to appropriately manage temporary drug holds during inter current illnesses while avoiding unnecessary permanent discontinuation. The lower rate of adverse event-related discontinuation in our structured nurse-delivered multi component group (8.4% vs. 15.2%) suggests that many discontinuations in conventional care may have been preventable through better anticipatory guidance. This is particularly important given that real-world persistence rates with SGLT2 inhibitors are sub optimal, with studies showing 30–40% discontinuation within the first year even among patients with clear indications [24, 25]. Third, systematic laboratory monitoring with rapid identification and management of electrolyte abnormalities or excessive eGFR decline allowed for proactive dose adjustments or temporary holds rather than reactive discontinuation after adverse events occurred. The higher monitoring compliance rates we observed (82.3% vs. 64.7%) facilitated earlier detection of potential safety concerns.

The integration of digital health tools and structured communication systems within structured nurse-delivered multi component group interventions has been shown to enhance diabetes management outcomes, and similar benefits likely accrued in our pathway through reminder systems and telephone follow-up protocols [26, 27]. The median of 5 nursing contacts per 6 months in our intervention group, compared to 1 contact in conventional care, reflects substantially greater patient engagement that likely contributed to improved medication persistence and adherence. While increased contact frequency requires additional nursing resources, the potential to prevent costly complications including dialysis initiation, hospitalizations, and cardiovascular events may justify this investment from both clinical and health economic perspectives. Although we did not directly quantify nursing time, implementation costs, reimbursement mechanisms, or downstream cost savings in this study, these factors represent critical targets for future multicenter implementation and health-economic evaluations assessing the scalability and payer sustainability of higher-intensity structured care pathways.

Our study complements and extends prior research on structured nurse-delivered multi component group chronic kidney disease management. Previous studies have demonstrated that diabetes specialist nurse interventions positively impact glycemic control and healthcare utilization through remote care delivery and intensified follow-up [28, 29]. Our findings extend these observations specifically to SGLT2 inhibitor management in CKD populations, demonstrating benefits on kidney function trajectories and hard clinical endpoints beyond metabolic control. The consistency of treatment effects across subgroups including patients with advanced CKD (eGFR < 30 mL/min/1.73 m²) and those with macroalbuminuria suggests that structured nurse-delivered multi component group pathways can be successfully implemented across the spectrum of diabetic kidney disease severity.

Several implementation considerations emerge from our experience. First, successful structured nurse-delivered multi component group SGLT2 inhibitor management requires adequate training and scope of practice for diabetes specialist nurses, including competence in kidney disease pathophysiology, volume assessment, and medication dose adjustment algorithms. Health systems approaches to structured nurse-delivered multi component group implementation should consider workforce development, infrastructure for virtual care delivery, and organizational support for expanded nursing roles [30]. Second, clear protocols defining the boundaries of nursing autonomy versus physician consultation are essential, with predefined thresholds for escalation including severe electrolyte abnormalities, acute kidney injury, or recurrent adverse events. Third, integration with electronic health record systems facilitating automated reminders, results tracking, and documentation is important for scalability and sustainability of structured nurse-delivered multi component group pathways beyond research settings.

This study has several strengths including the real-world design reflecting routine clinical practice, substantial sample size adequate for detecting clinically meaningful effects, comprehensive covariate collection enabling robust propensity score adjustment, and systematic outcome ascertainment through electronic data sources minimizing recall or ascertainment bias. The single-center setting allowed for consistent pathway implementation with well-trained nursing staff and standardized protocols. The use of propensity score methods represents contemporary approaches to strengthening causal inference from observational data by mimicking attributes of randomized trials through covariate balancing, though residual confounding from unmeasured factors remains possible [31, 32]. Importantly, the observed associations do not reflect autonomous nurse prescribing but rather a collaborative care model in which nurses operationalized protocolized monitoring and follow-up under physician supervision.

### Limitations

Important limitations must be acknowledged. The retrospective design precludes definitive causal inference despite propensity score adjustment, as unmeasured confounders including patient motivation, health literacy, or provider practice patterns may have influenced both pathway participation and outcomes. In addition, the intervention was implemented as a multi component care delivery bundle rather than a single discrete exposure. Consequently, we cannot determine which individual elements such as increased contact frequency, intensified laboratory monitoring, adherence reminders, patient education, or medication titration support were most responsible for the observed associations. The improved outcomes may reflect synergistic effects of these implementation supports in enhancing medication persistence, timely laboratory surveillance, and early management of adverse events. Selection bias and residual confounding may have occurred if patients with greater motivation, higher health literacy, better access to care, or stronger social support were more likely to enroll in and remain engaged with the pathway. Although proxy indicators of healthcare engagement and access were incorporated into the propensity score model, these measures cannot fully capture latent factors such as motivation, digital literacy, transportation barriers, or provider preference. Consequently, the observed associations may partially reflect differences in patients’ capacity to engage in intensive longitudinal care rather than the independent effect of the pathway components themselves.

Additional limitations include the relatively short follow-up duration of 6–24 months, which may not capture longer-term effects on end-stage kidney disease or mortality. Longer follow-up studies are needed to confirm sustained benefits and assess whether improved kidney function trajectories translate to clinically meaningful delays in dialysis or transplantation needs. Some outcomes including medication adherence relied on self-report or pharmacy dispensing records rather than direct observation, potentially introducing measurement error. The study was not powered to detect subgroup differences, so absence of statistically significant interactions should not be over-interpreted as definitive evidence of effect homogeneity. Finally, we could not assess cost-effectiveness given limitations in capturing complete healthcare utilization and cost data from our single-center EMR system, though future economic evaluations incorporating both intervention costs and downstream savings from prevented complications would be valuable.

An important next step is to assess the scalability and sustainability of this structured, nurse delivered multicomponent pathway across other hospitals and healthcare settings. Multi-center implementation studies with longer follow-up durations would allow evaluation of generalizability, durability of effects, and longer-term outcomes such as progression to end-stage kidney disease, kidney replacement therapy initiation, mortality, and healthcare utilization. Pragmatic designs, including cluster-randomized or stepped-wedge implementation trials, may be particularly well suited to support staged roll-out while accounting for site-level differences in staffing models, scope-of-practice regulations, and baseline SGLT2 inhibitor adoption.

Potential role of pharmacist led models. In addition to nurse-delivered pathways, pharmacist led or pharmacist-integrated care models may represent an effective alternative strategy to increase appropriate SGLT2 inhibitor use. Clinical pharmacists possess expertise in medication reconciliation, dose optimization, drug drug interaction management, and patient counseling, all of which align closely with the core elements of the implementation bundle evaluated in this study. Under collaborative practice agreements or physician oversight, pharmacist led protocols could similarly support standardized initiation, monitoring, and adherence reinforcement for SGLT2 inhibitors. Future comparative implementation studies evaluating nurse led, pharmacist led, or hybrid team based models may help identify the most efficient and scalable approaches across different healthcare settings. Because the pathway represents a bundled implementation strategy, the observed benefits cannot be attributed to nursing leadership per se but rather to the combined effects of intensified monitoring, standardized protocols, and increased patient engagement, which together constitute the higher-intensity care model. Although we performed fixed-window (landmark) slope analyses to reduce sensitivity to differential measurement intensity, residual informative observation bias cannot be fully excluded in retrospective data where visit timing may correlate with unmeasured clinical status.

### Conclusion

In conclusion, in this real-world retrospective study, participation in a structured, nurse-delivered multi component SGLT2 inhibitor management pathway was associated with improved kidney function trajectories and fewer adverse kidney outcomes among patients with T2DM and CKD. These findings reflect the combined effects of pharmacologic therapy and enhanced implementation supports including frequent contact, structured monitoring, and adherence reinforcement rather than the isolated effect of any single pathway component. The results support the feasibility and potential effectiveness of structured care delivery models to bridge the evidence practice gap in SGLT2 inhibitor use. Future cluster randomized and implementation studies across diverse healthcare settings will be required to establish causal effects and to disentangle the relative contributions of individual pathway components.

## Supplementary Information

Below is the link to the electronic supplementary material.

Supplementary Material 1

Supplementary Material 2

## Data Availability

All the data analysed/generated during this study will be available from corresponding author upon reasonable request.

## Abbreviations

CKD: Chronic kidney disease
eGFR: Estimated glomerular filtration rate
EMR: Electronic medical record
ESKD: End-stage kidney disease
HR: Hazard ratio
NIS: Nursing information system
PS: Propensity score
SGLT2: Sodium–glucose cotransporter-2
T2DM: Type 2 diabetes mellitus
