# Supplementary material for: Impact of a structured nurse-delivered multi component SGLT2 inhibitor initiation and optimization pathway on kidney function and clinical outcomes in type 2 diabetes and chronic kidney disease: a real-world retrospective study
Source: BMC Nephrol. 2026 Mar 14;27:275. doi: 10.1186/s12882-026-04842-z (PMC13134205; doi:10.1186/s12882-026-04842-z)
Supplement: Supplementary file 1 — Supplementary Material 1 [file 12882_2026_4842_MOESM1_ESM.docx]

**Figure.S1 Distribution of eGFR measurement timing by study group**


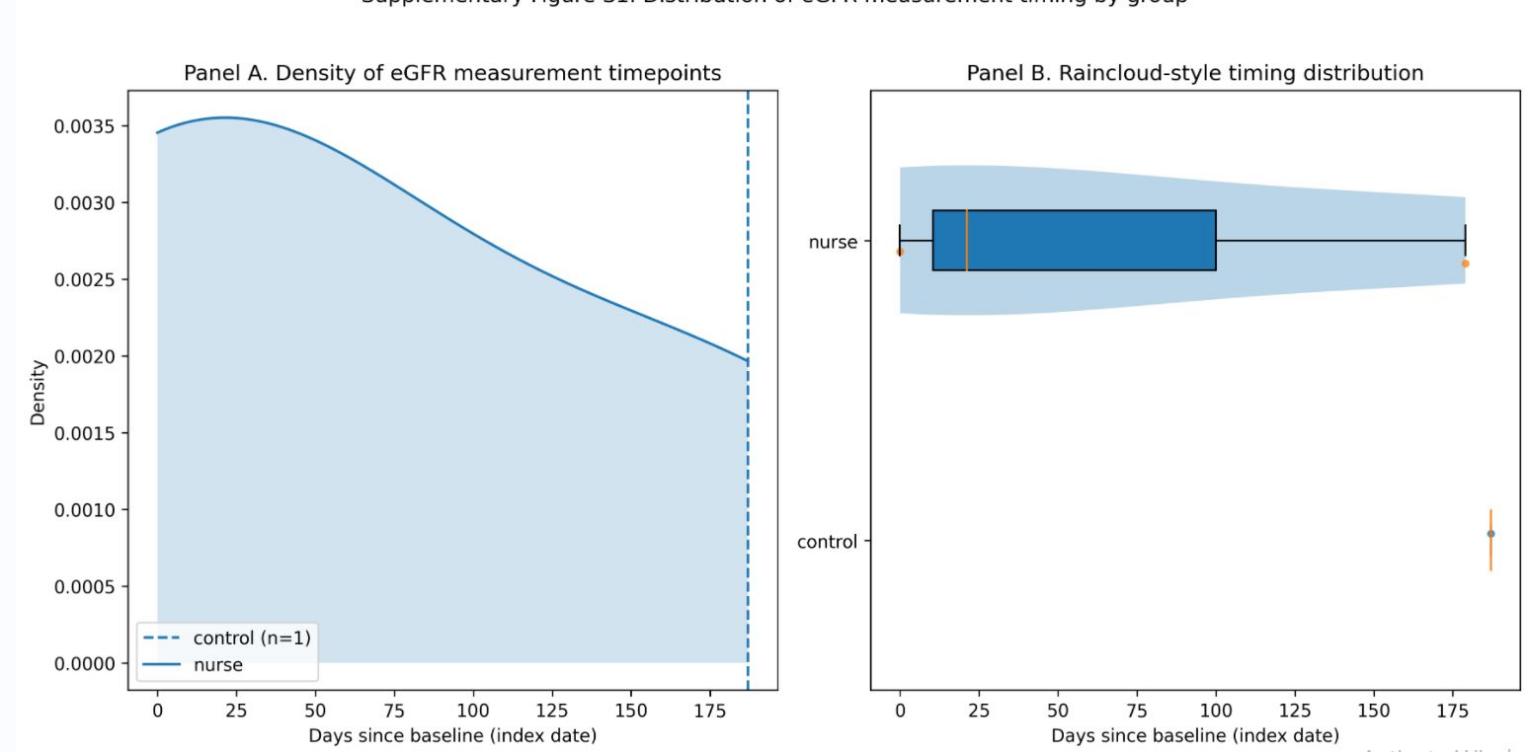


Distribution of eGFR measurement timing relative to baseline by study group. Density/raincloud plots illustrate higher measurement intensity in the structured pathway group, consistent with protocolized follow-up.
