# Supplementary material for: Impact of a structured nurse-delivered multi component SGLT2 inhibitor initiation and optimization pathway on kidney function and clinical outcomes in type 2 diabetes and chronic kidney disease: a real-world retrospective study
Source: BMC Nephrol. 2026 Mar 14;27:275. doi: 10.1186/s12882-026-04842-z (PMC13134205; doi:10.1186/s12882-026-04842-z)
Supplement: Supplementary file 2 — Supplementary Material 2 [file 12882_2026_4842_MOESM2_ESM.docx]

| Group | Patients | eGFR measures/patient (median, IQR) | Time to first post-baseline eGFR (days), median (IQR) |
| --- | --- | --- | --- |
| control | 1 | 1 (1–1) | 187 (187–187) |
| nurse | 1 | 3 (3–3) | 0 (0–0) |

# Supplementary Table S1. Observation process summaries

This table summarizes laboratory measurement frequency and timing and is not intended as an outcome comparison.

# Supplementary Table S2. Landmark eGFR slope sensitivity analyses (fixed windows)

| Group | N with 6-month slope | Slope 0–6 months (mean ± SD) | N with 12-month slope | Slope 0–12 months (mean ± SD) |
| --- | --- | --- | --- | --- |
| control | 1 | -4.06 | 0 |  |
| nurse | 1 | -4.49 | 0 |  |

Landmark slopes used standardized nearest-to-landmark selection (6 months: 180±30 days; 12 months: 365±45 days) to reduce sensitivity to differential measurement frequency.
